# Primary versus elective percutaneous transluminal angioplasty with stenting for symptomatic intracranial atherosclerotic stenosis: a 18-centre retrospective cohort study using the pEGASUS-HPC stent

**DOI:** 10.1093/esj/aakag091

**Published:** 2026-07-31

**Authors:** Ali Khanafer, Ahmed Ayad, Mohammad Almohammad, Pablo Albiña-Palmarola, Ivan Lylyk, Florian Eff, Islambek Mussabekov, Alexander Sirakov, Marie-Sophie Schüngel, Tolga Tonguc, Donald Lobsien, Johannes Rückel, Aikaterini Anastasiou, Muhammad AlMatter, Zakarya Ali, Mete Dadak, Kamran Hajiyev, Mehdi Allouche, Philipp von Gottberg, Sebastian Johannes Müller, Jonas Schüssler, Shinya Hagiwara, Abdallah Aburub, Marat Sarshayev, Hansjörg Bäzner, Marios Psychogios, Thomas Liebig, José E Cohen, Stephan Felber, Joachim Klisch, Franziska Dorn, Stanimir Sirakov, Markus Holtmannspötter, Stefan Schob, Mynzhylky Berdikhojayev, André Kemmling, Michael Forsting, Pedro Lylyk, Rene Chapot, Hans Henkes

**Affiliations:** Neuroradiology, Klinikum Stuttgart Katharinenhospital, Stuttgart, Germany; Diagnostic and Interventional Radiology and Neuroradiology, Alfried-Krupp-Hospital, Essen, Germany; Department of Neuroradiology, University Marburg, Marburg, Germany; Neuroradiology, Klinikum Stuttgart Katharinenhospital, Stuttgart, Germany; Department of Neuroradiology, Clínica La Sagrada Familia, Buenos Aires, Argentina; Department of Neuroradiology, Nuremberg Hospital, Paracelsus Medical University, Nuremberg, Germany; Medical Centre Hospital of the President’s Affairs Administration of the Republic of Kazakhstan, Astana City, Kazakhstan; Astana Medical University, Astana, Kazakhstan; Radiology Department, University Hospital St Ivan Rilski, Sofia, Bulgaria; Klinikum St. Georg, Klinik für Neuroradiologie, Leipzig, Germany; Department of Neuroradiology, University Hospital Bonn, Bonn, Germany; Klinikum Chemnitz gGmbH, Institut Für Radiologie Und Neuroradiologie, Chemnitz, Germany; Institute for Diagnostic and Interventional Neuroradiology, LMU University Hospital, LMU Munich, Munich, Germany; Department of Diagnostic and Interventional Neuroradiology, University Hospital Basel, Basel, Switzerland; Radiology and Nuclear Medicine, Schwarzwald-Baar Hospital, Villingen-Schwenningen, Germany; Central Institute for Diagnostic and Interventional Radiology Neuroradiology and Nuclear Medicine, Offenbach am Main, Germany; Department of Radiology, St Vincenz Hospital Paderborn, Paderborn, Germany; Neuroradiology, Klinikum Stuttgart Katharinenhospital, Stuttgart, Germany; Neuroradiology, Klinikum Stuttgart Katharinenhospital, Stuttgart, Germany; Neuroradiology, Klinikum Stuttgart Katharinenhospital, Stuttgart, Germany; Clinic of Neuroradiology, University Magdeburg, Magdeburg, Germany; Diagnostic and Interventional Radiology and Neuroradiology, Alfried-Krupp-Hospital, Essen, Germany; Diagnostic and Interventional Radiology and Neuroradiology, Alfried-Krupp-Hospital, Essen, Germany; Department of Neuroradiology, University Marburg, Marburg, Germany; National Hospital of the Medical Center of the Presidential Administration of the Republic of Kazakhstan, Almaty City, Kazakhstan; Klinik für Neurologie, Klinikum Stuttgart Katharinenhospital, Stuttgart, Germany; Department of Diagnostic and Interventional Neuroradiology, University Hospital Basel, Basel, Switzerland; Institute for Diagnostic and Interventional Neuroradiology, LMU University Hospital, LMU Munich, Munich, Germany; Neurosurgery, Hadassah-Hebrew Univ Med Ctr, Jerusalem, Israel; Department of Diagnostic and Interventional Radiology Neuroradiology, Gemeinschaftsklinikum Koblenz Mayen, Koblenz, Germany; Clinic of Neuroradiology, Health and Medical University, Helios General Hospital Erfurt, Germany; Department of Neuroradiology, University Hospital Bonn, Bonn, Germany; Radiology Department, University Hospital St Ivan Rilski, Sofia, Bulgaria; Department of Neuroradiology, Nuremberg Hospital, Paracelsus Medical University, Nuremberg, Germany; Klinikum St. Georg, Klinik für Neuroradiologie, Leipzig, Germany; Department of Neuroradiology, University Hospital Halle, Halle, Germany; National Hospital of the Medical Center of the Presidential Administration of the Republic of Kazakhstan, Almaty City, Kazakhstan; Department of Neuroradiology, University Marburg, Marburg, Germany; Department of Diagnostic and Interventional Radiology and Neuroradiology, Schön Klinik Neustadt, Neustadt in Holstein, Germany; Institut für Diagnostische und Interventionelle Radiologie und Neuroradiologie, Universitätsklinikum Essen, Essen, Germany; Department of Neuroradiology, Clínica La Sagrada Familia, Buenos Aires, Argentina; Diagnostic and Interventional Radiology and Neuroradiology, Alfried-Krupp-Hospital, Essen, Germany; Neuroradiology, Klinikum Stuttgart Katharinenhospital, Stuttgart, Germany; Institut für Diagnostische und Interventionelle Radiologie und Neuroradiologie, Universitätsklinikum Essen, Essen, Germany

**Keywords:** acute ischaemic stroke, intracranial angioplasty, intracranial atherosclerotic stenosis, intracranial stenting, pEGASUS-HPC, percutaneous transluminal angioplasty

## Abstract

**Introduction:**

To describe the safety and effectiveness of percutaneous transluminal angioplasty with pEGASUS-HPC stenting (PTAS) for symptomatic intracranial atherosclerotic stenosis (sICAS) and compare primary versus elective use.

**Patients and methods:**

We retrospectively included consecutive adults with sICAS ≥50% treated with pEGASUS-HPC PTAS at 18 neurovascular centres (May 2021–February 2025). Primary PTAS was defined as treatment during non-large-vessel-occlusion acute ischaemic stroke (AIS); Elective PTAS was performed at least 7 days after the qualifying ischaemic presentation for recurrent symptoms or haemodynamic compromise despite intensive medical therapy. Outcomes included technical success (post-treatment stenosis ≤50% without intraprocedural complications), in-hospital major complications (intracerebral haemorrhage, AIS or death), restenosis and functional status, measured by the modified Rankin Scale (mRS) at last follow-up.

**Results:**

Among 141 patients (Primary 71; Elective 70), median post-treatment stenosis was 23.0%; technical success was 94.3% (90.1% vs 98.6%; *P* = .063). In-hospital major complications occurred in 7.1% (12.7% Primary vs 1.4% Elective; *P* = .017), driven by intraprocedural in-stent thrombosis (9.9% vs 0%; *P* = .013). At last follow-up (median 9 months), restenosis ≥50% was observed in 14.3% (7.8% vs 19.7%; *P* = .10) and mRS <3 in 84.7% (82.1% vs 87.1%; *P* = .61). New diffusion-weighted imaging lesions were seen in 39.2% of 79 patients with early MRI, without group differences.

**Discussion:**

In this multicentre cohort, pEGASUS-HPC PTAS provided substantial luminal gain, moderate restenosis and good mid-term functional outcomes overall. Elective use in medically refractory sICAS was associated with very low major complication rates, whereas primary use during AIS carried significantly higher thrombotic and haemorrhagic risk.

## Graphical Abstract

**Graphical Abstract:**
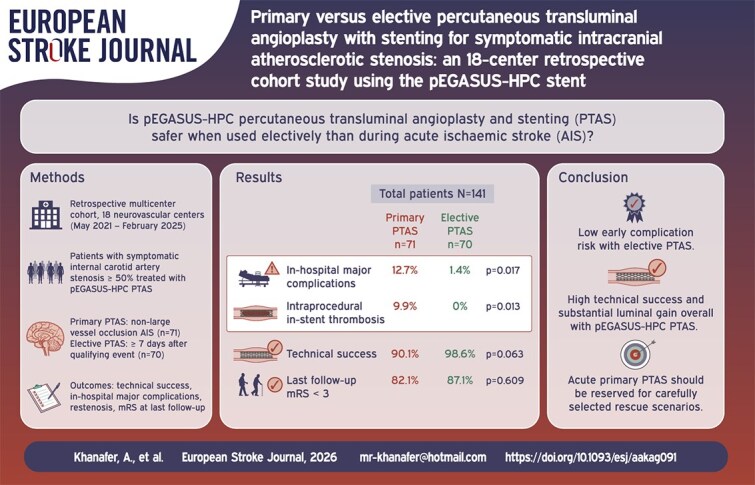

## Introduction

Symptomatic intracranial atherosclerotic stenosis (sICAS) is a major cause of acute ischaemic stroke (AIS) and transient ischaemic attack (TIA) worldwide and carries a high risk of recurrent events despite contemporary secondary prevention. Randomised clinical trials (RCTs) such as the Warfarin–Aspirin Symptomatic Intracranial Disease (WASID) trial established antiplatelet-based medical therapy as the standard of care, showing that warfarin offered no benefit and increased bleeding and death in patients with sICAS.^1^ Subsequent trials of percutaneous transluminal angioplasty and stenting (PTAS) with first-generation devices (Wingspan and balloon-expandable stents) in addition to intensive medical therapy (IMT) did not improve outcomes and were associated with excess early stroke compared with IMT alone.^2^^,^^3^ On the basis of these data, current guidelines recommend IMT as first-line treatment and advise against routine intracranial stenting as initial therapy in most patients with sICAS.^4^

Despite this, a clinically important subgroup continues to experience recurrent ischaemic events or disabling haemodynamic symptoms under guideline-concordant therapy.^4^ In this context, endovascular strategies such as balloon angioplasty (submaximal or maximal) and stenting have been explored as rescue options in carefully selected, medically refractory patients. Prospective series and, more recently, the BASIS RCT of submaximal angioplasty have suggested acceptable periprocedural risks and potential efficacy in experienced centres, particularly when combined with IMT^5^^,^^6^; however, systematic reviews and meta-analyses of PTAS have reported high technical success but non-negligible rates of stroke and haemorrhage, with persistent uncertainty regarding optimal timing, device selection and patient profiles.^7^^,^^8^

Newer neurovascular implants with surface-modifying technologies have been developed to reduce device thrombogenicity and potentially allow more flexible antiplatelet regimens. Hydrophilic polymer coating (HPC) technology, initially used on the p48 flow diverter, reduced thrombin generation and platelet activation in vitro compared with uncoated devices,^9^ and early clinical experience with HPC-coated devices in aneurysm treatment suggested good deliverability and low acute thromboembolic complication rates.^10–12^ A recent multicentre study of the pEGASUS-HPC stent in acute sICAS reported high technical success and encouraging short-term outcomes but was limited to the acute setting and modest sample size.^10^ Overall, clinical data on HPC-coated intracranial stents for sICAS remain sparse and largely confined to single-centre or early multicentre series.^7^^,^^10^^,^^13^

The pEGASUS-HPC stent (Phenox GmbH, Bochum, Germany) is a low-profile, self-expanding, open-cell nitinol stent with an antithrombogenic HPC designed for intracranial aneurysms, dissections and atherosclerotic stenosis. Building on increasing use of this device for sICAS in many neurovascular centres, both as primary treatment during AIS and as elective PTAS after failure of IMT, there is a need for larger, systematically collected real-world data. We therefore conducted a retrospective, multicentre cohort study across 18 neurovascular centres to describe procedural characteristics, technical success, periprocedural complications and short- to mid-term angiographic and clinical outcomes in adults with high-grade sICAS treated with pEGASUS-HPC PTAS, with a prespecified comparison between Primary and Elective PTAS strategies.

## Patients and methods

### Study design and setting

We conducted a retrospective multicentre cohort study across 18 neurovascular centres, including consecutive patients treated with pEGASUS-HPC PTAS between May 2021 and February 2025. The study followed STROBE recommendations and was approved by the local institutional review board at each site with waiver of informed consent, in accordance with national regulations for retrospective analyses.

### Patients

Eligible patients were adults with sICAS ≥50% on digital subtraction angiography (DSA), with percent stenosis measured using the WASID method,^14^ and a qualifying TIA or AIS within 90 days attributable to the target lesion.^4^ Target-lesion eligibility was based on symptomatic intracranial large-artery atherosclerotic stenosis rather than on a prespecified arterial segment; both anterior- and posterior-circulation lesions were included when considered suitable for PTAS by the treating team. Attribution of the qualifying event to the target lesion was based on local clinical and angiographic assessment, including infarct topography, vascular territory, stenosis severity, recurrent symptoms and haemodynamic information when available. Patients were classified a priori into 2 groups:

- Primary PTAS: performed during AIS in the absence of large-vessel occlusion (LVO) requiring mechanical thrombectomy, restricted to patients with an NIHSS score ≥ 7. These cases represented acute symptomatic high-grade stenoses considered responsible for a disabling non-LVO ischaemic presentation, usually because of flow limitation, recurrent or progressive symptoms, or haemodynamic compromise despite acute medical management. They were not rescue stenting procedures performed after thrombectomy for embolic LVO.
- Elective PTAS: planned stenting performed at least 7 days after the qualifying ischaemic presentation for symptomatic sICAS with recurrent ischaemic symptoms or haemodynamic compromise despite IMT. These procedures were not performed as emergency treatment during the index acute ischaemic presentation; patients either had no ongoing symptoms at treatment or had only mild/non-disabling symptoms.

Exclusion criteria were non-atherosclerotic intracranial stenosis, treatment performed as part of mechanical thrombectomy for LVO, incomplete core procedural data or absence of any post-procedure clinical follow-up.

### Device and procedure

All procedures used the pEGASUS-HPC stent. Access route, lesion preparation (eg, single or sequential balloon pre-dilation with or without drug-coated balloons), stent diameter and length selection and post-dilation were at operator discretion according to local practice. Proximal and distal reference vessel diameters were measured on DSA; stent oversizing at each landing zone was derived from nominal stent diameter and the corresponding vessel diameter. Periprocedural antiplatelet management followed institutional protocols.

### Variables and outcomes

Baseline demographics, vascular risk factors, index event type, lesion location and side, reference vessel diameters and percent stenosis before and after stenting were collected in standardised case report forms. When available, diffusion-weighted MRI (DWI) within the index hospitalisation was reviewed. The primary procedural outcome was technical success, defined a priori as post-treatment stenosis ≤50% and no intraprocedural complications (sensitivity: residual ≤30%). Intraprocedural complications included in-stent thrombosis, distal embolism, angiographically evident dissection, vessel perforation and deployment-related technical problems, defined as unsuccessful stent opening, stent malposition, stent migration or inability to deploy or retrieve the device as intended. The primary in-hospital safety outcome was major complications, defined as any intracranial haemorrhage (ICH), AIS or death before discharge. Other in-hospital events (eg, TIA without new infarction) were recorded descriptively. Follow-up (FU) assessments were captured as part of routine clinical care and recorded as months from the index procedure. We summarise outcomes at nominal FU visits and at the last available FU. Functional status was assessed with the modified Rankin Scale (mRS); we report mRS < 3 (functional independence) and a 3-level mRS shift (ΔmRS: Improved, Stable, Worsened), defined as the difference between the last-available and baseline mRS. Baseline mRS was defined as the mRS recorded immediately before the index PTAS procedure. Accordingly, in Primary PTAS patients, baseline mRS generally reflected the acute stroke presentation, whereas in Elective PTAS patients it reflected preprocedural functional status after the qualifying event and any interval recovery. Therefore, ΔmRS analyses were interpreted descriptively and were not used to infer differential functional recovery between treatment contexts. Deaths were coded as mRS = 6 from the time they occurred and carried forward to the last available FU; otherwise, analyses were conducted on an observed-case basis without imputation. Angiographic restenosis at FU was recorded according to site practice and typically defined as ≥50% luminal narrowing of the treated segment on vascular imaging. Angiographic measurements, DWI findings, restenosis and clinical outcomes were assessed according to local site practice and were not adjudicated by an independent core laboratory or central clinical-events committee.

### Statistical analysis

Analyses were descriptive with group comparisons between Primary and Elective PTAS. The Primary versus Elective PTAS comparison was defined a priori for the present analysis and was intended to describe outcome patterns across 2 real-world treatment contexts; because treatment context was not randomised and indications differed by design, between-group comparisons were interpreted descriptively rather than as causal estimates. Continuous and ordinal variables are summarised as median and IQR and compared using Wilcoxon rank-sum tests. Categorical variables are presented as counts and percentages and compared using Pearson’s χ^2^ test or Fisher’s exact test, as appropriate. Three-level mRS shift distributions (Improved/Stable/Worsened) were compared using Pearson’s χ^2^; per-category 2 × 2 contrasts were explored with Fisher’s exact test. All *P*-values are 2-sided with α = .05 and no adjustment for multiple comparisons, reflecting the exploratory, descriptive nature of the study. All analyses were performed in Stata 19.5 (StataCorp, College Station, TX).

## Results

### Study population and baseline characteristics

From May 2021 to February 2025, 141 consecutive adults with sICAS treated with pEGASUS-HPC PTAS at 18 centres from 5 countries were included: 71 in the Primary PTAS group (during non-LVO AIS) and 70 in the Elective PTAS group (planned treatment at least 7 days after the qualifying ischaemic presentation for recurrent symptoms or haemodynamic compromise despite IMT). Median age was 68.0 years (IQR 58.5–76.0, mean 66.8 ± 12.4) and 48/141 patients (34.0%) were female. Vascular risk factors were frequent, including hypertension in 115/141 (81.6%), diabetes mellitus in 61/141 (43.3%), dyslipidaemia in 73/141 (51.8%), current smoking in 31/141 (22.0%) and coronary artery disease in 58/141 (41.1%). The distribution of comorbidities was similar between groups (all *P* > .05), as shown in Table 1.

**Table 1:** Baseline characteristics according to treatment modality.

Baseline characteristics	Overall, *n* = 141	Primary PTAS (AIS), *n* = 71	Elective PTAS (Symptomatic), *n* = 70	*P*-value
**Age, years (median [IQR])**	68.0 [58.5, 76.0]	67.0 [57.0, 76.0]	69.0 [60.0, 76.0]	.488
**Sex**				.214
**Female**	48 (34.0)	28 (39.4)	20 (28.6)	
**Male**	93 (66.0)	43 (60.6)	50 (71.4)	
**Obesity**	32 (22.7)	16 (22.5)	16 (22.9)	1.000
**Hypertension**	115 (81.6)	54 (76.1)	61 (87.1)	.128
**Type 2 diabetes**	61 (43.3)	29 (40.9)	32 (45.7)	.612
**Dyslipidaemia**	73 (51.8)	32 (45.1)	41 (58.6)	.130
**Current smoking**	31 (22.0)	11 (15.5)	20 (28.6)	.070
**Coronary artery disease**	58 (41.1)	26 (36.6)	32 (45.7)	.307
**Previous cerebrovascular event**				**.001**
**Territorial stroke**	54 (38.3)	34 (47.9)	20 (28.6)	
**Lacunar stroke**	51 (36.2)	21 (29.6)	30 (42.9)	
**TIA**	29 (20.6)	9 (12.7)	20 (28.6)	
**Asymptomatic**	3 (2.1)	3 (4.2)	0 (0.0)	
**Unknown**	4 (2.8)	4 (5.6)	0 (0.0)	
**Recurrent symptoms**	81 (57.9)	29 (41.4)	52 (74.3)	**<.001**
**Baseline mRS**				.547
**mRS < 3**	109 (77.3)	53 (74.7)	56 (80.0)	
**mRS ≥3**	32 (22.7)	18 (25.4)	14 (20.0)	

Data are median [IQR] or *n* (%). *P*-values: continuous variables by Mann–Whitney U (exact when available); categorical variables by Fisher’s exact test (r × c extension for multi-category variables). Percentages within Primary PTAS and Elective PTAS are column percentages; the Overall column uses the available denominator for each variable (minor missingness in “Age” and “Recurrent symptoms”: 1 case). Abbreviations: AIS = acute ischaemic stroke; mRS = modified Rankin Scale; PTAS = percutaneous transluminal angioplasty and stenting; TIA = transient ischaemic attack. Bold values indicate statistically significant differences (P < .05).

The pattern of prior cerebrovascular events differed between Primary and Elective PTAS (global *P* = .001), with more territorial strokes in the Primary group and more TIAs and lacunar strokes in the Elective group (Table 1). Baseline functional status was comparable, with functional independence (mRS < 3) in 109/141 patients (77.3%) overall (74.6% Primary vs 80.0% Elective; *P* = .547). Recurrent ischaemic symptoms before the index procedure were present in 81/140 patients (57.9%) and were significantly more frequent in the Elective PTAS group (74.3% [52/70] vs 41.4% [29/70]; *P* < .001; Table 1). The lacunar stroke category referred to the recorded infarct pattern/topography rather than to a presumed isolated small-vessel mechanism; cases were included only when the treating team considered the event clinically attributable to the target intracranial stenosis.

### Lesion characteristics and procedural details

Lesions were most commonly located in the M1 segment (46/141, 32.6%), followed by V4 (25/141, 17.7%), basilar artery (24/141, 17.0%) and supraclinoid (17/141, 12.1%) and cavernous internal carotid artery (12/141, 8.5%), with similar anatomical distribution across groups (*P* = .564; see Table 2). Petrous ICA lesions accounted for 10/141 cases (7.1%) and are reported separately because classification of this skull-base ICA segment varies across ICAS studies. Pre-treatment stenosis was severe, with a median of 80.0% [IQR 70.0–87.0] overall and comparable values in the Primary and Elective PTAS cohorts (81.0% [70.0–90.0] vs 80.0% [70.0–86.0]; *P* = .206).

**Table 2:** Lesion characteristics and procedural details by treatment group.

Variable /category	Overall (*N* = 141)	Primary PTAS (*n* = 71)	Elective PTAS (*n* = 70)	*P*-value
**Lesion and vessel metrics**				
**Location**				.564
** Petrous ICA**	10 (7.1)	4 (5.6)	6 (8.6)	
** Cavernous ICA**	12 (8.5)	7 (9.9)	5 (7.1)	
** Supraclinoid ICA**	17 (12.1)	6 (8.5)	11 (15.7)	
** M1**	46 (32.6)	24 (33.8)	22 (31.4)	
** M2**	4 (2.8)	3 (4.2)	1 (1.4)	
** V3**	2 (1.4)	1 (1.4)	1 (1.4)	
** V4**	25 (17.7)	10 (14.1)	15 (21.4)	
** BA**	24 (17.0)	15 (21.1)	9 (12.9)	
** A1**	1 (0.7)	1 (1.4)	0 (0.0)	
**Side**				.308
** Left**	70 (49.7)	34 (47.9)	36 (51.4)	
** Right**	46 (32.6)	21 (29.6)	25 (35.7)	
** Midline**	25 (17.7)	16 (22.5)	9 (12.9)	
**Pre-treatment stenosis (%)**	80.0 [70.0, 87.0]	81.0 [70.0, 90.0]	80.0 [70.0, 86.0]	.206
**Proximal vessel diameter (mm)**	2.8 [2.2, 3.6]	2.7 [2.1, 3.5]	3.0 [2.2, 3.8]	.193
**Distal vessel diameter (mm)**	2.5 [2.1, 3.2]	2.4 [1.9, 3.1]	2.8 [2.2, 3.3]	**.010**
**Procedural details**				
**Arterial access**				.097
** Femoral**	132 (93.6)	69 (97.2)	63 (90.0)	
** Radial**	9 (6.4)	2 (2.8)	7 (10.0)	
**No. of implanted stents (first session)**				.063
** 1**	133 (94.3)	64 (90.1)	69 (98.6)	
** 2**	8 (5.7)	7 (9.9)	1 (1.4)	
**Stent diameter (mm)**	3.5 [3.5, 4.5]	3.5 [3.5, 4.5]	3.5 [3.5, 4.5]	.812
**Stent length (mm)**	20.0 [15.0, 20.0]	20.0 [15.0, 20.0]	20.0 [15.0, 20.0]	.918
**Proximal stent oversizing (%)**	34.6 [9.8, 66.7]	34.6 [16.7, 75.0]	32.4 [4.7, 66.7]	.171
**Distal stent oversizing (%)**	45.8 [18.4, 84.2]	59.1 [29.6, 95.7]	38.2 [9.4, 66.7]	**.007**
**Balloon pre-dilation**	137 (97.2)	70 (98.6)	67 (95.7)	.366
**Second balloon for pre-dilation**	50 (35.5)	14 (19.7)	36 (51.4)	**<.001**
**DEB for pre-dilation**	29 (20.6)	4 (5.6)	25 (35.7)	**<.001**
**Post-dilation**	10 (7.1)	7 (9.9)	3 (4.3)	.326
**DEB for post-dilation**	1 (10.0)	0 (0.0)	1 (33.3)	.300
**Antiplatelet scheme (prior treatment)**				**<.001**
** SAPT**	34 (30.4)	23 (54.8)	11 (15.7)	
** DAPT**	78 (69.6)	19 (45.2)	59 (84.3)	
**Postprocedural antiplatelet scheme**				**.001**
** SAPT**	14 (9.9)	13 (18.3)	1 (1.4)	
** DAPT**	127 (90.1)	58 (81.7)	69 (98.6)	

Values are median [IQR] or *n* (column %). *P*-values: Mann–Whitney U for continuous; Pearson χ^2^ or Fisher’s exact as appropriate for categorical (small expected counts). Percentages use each column’s non-missing denominator; “Overall” uses the variable’s non-missing denominator. Missing values: post-dilation balloon type (*n* = 131), DEB for post-dilation (*n* = 131), antiplatelet premedication scheme (*n* = 29), antiplatelet loading drugs (*n* = 59); all other variables 0. Post-dilation balloon type and DEB for post-dilation are reported only among cases with post-dilation (overall *n* = 10; Primary 7, Elective 3). Abbreviations: AIS = acute ischaemic stroke; DEB = drug-eluting balloon; ICH = intracerebral haemorrhage; *N* = number; PTAS = percutaneous transluminal angioplasty and stenting; SD = standard deviation; TIA = transient ischaemic attack. Bold values indicate statistically significant differences (P < .05).

Stent dimensions were similar between groups, with a median nominal diameter of 3.5 mm (3.5–4.5) and length of 20.0 mm (15.0–20.0). Proximal stent oversizing was 34.6% (9.8–66.7) overall, without significant group difference (*P* = 0.171), whereas distal oversizing was greater in Primary PTAS (59.1% [29.6–95.7] vs 38.2% [9.4–66.7]; *P* = 0.007). Most procedures used transfemoral access; radial access was employed in 9/141 interventions (6.4%), more often in Elective PTAS (10.0% vs 2.8%; *P* = .097). No access-site complications were reported. Pre-dilation was performed in 137/141 cases (97.2%), with similar overall frequency between groups, but different balloon strategies: NeuroSpeed was used more often in Primary PTAS (42.9% vs 13.4%), whereas pITA and drug-eluting balloons (SeQuent/Elutax) were more frequent in Elective PTAS (Table S1). Two stents were implanted in the first session in 8/141 cases (5.7%), more often in Primary PTAS (9.9% vs 1.4%; *P* = .063). Post-dilation was performed in 10/141 (7.1%). Drug-eluting balloons were used for pre-dilation in 29/141 patients (20.6%), substantially more often in Elective PTAS (35.7% vs 5.6%; *P* < .001; Table 2).

Periprocedural antiplatelet regimens reflected local protocols and differed between groups (Table S2). Patients undergoing Elective PTAS were more often on established dual antiplatelet therapy (DAPT) before the procedure, whereas Primary PTAS cases more frequently received acute loading in the emergency setting. Postprocedural maintenance most commonly consisted of DAPT for several months, followed by single antiplatelet therapy (SAPT), with only a small minority of patients treated with SAPT alone from the outset. In an exploratory analysis restricted to the Primary PTAS cohort, postprocedural SAPT (*n* = 13) was compared with DAPT (*n* = 58; Table S3). Residual stenosis was similar between groups, and no statistically significant differences were detected in intraprocedural in-stent thrombosis, distal embolism, postprocedural ischaemic events, new DWI lesions, restenosis or in-hospital major complications, although in-stent thrombosis and new DWI lesions were numerically more frequent with SAPT.

### Immediate angiographic and in-hospital outcomes

Post-treatment stenosis was reduced to a median of 23.0% (IQR 16.0–30.0), with similar values in Primary (25.0% [17.0–30.0]) and Elective PTAS (22.0% [15.0–30.0]; *P* = .565). The median absolute reduction in stenosis was 55.0% (IQR 45.0–65.0) overall and did not differ between groups (55.0% [46.0–65.0] vs 54.1% [45.0–65.0]; *P* = .814; see Table 3).

**Table 3:** Periprocedural and in-hospital outcomes, DWI lesions, restenosis and follow-up outcomes.

Outcome	Overall	Primary PTAS (AIS), *n* = 71	Elective PTAS (Symptomatic), *n* = 70	*P*-value
**Post-treatment stenosis (%)**	23.0 [16.0, 30.0]	25.0 [17.0, 30.0]	22.0 [15.0, 30.0]	.565
**Stenosis reduction (% post–pre)**	55.0 [45.0, 65.0]	55.0 [46.0, 65.0]	54.1 [45.0, 65.0]	.814
**Technical complications with stent deployment**	2/141 (1.4%)	2/71 (2.8%)	0/70 (0.0%)	.496
**Intraprocedural in-stent thrombosis**	7/141 (5.0%)	7/71 (9.9%)	0/70 (0.0%)	**.013**
**Intraprocedural distal embolism**	0/141 (0.0%)	0/71 (0.0%)	0/70 (0.0%)	N/A
**Intraprocedural arterial dissection**	1/141 (0.7%)	1/71 (1.4%)	0/70 (0.0%)	1.000
**Technical success (residual stenosis ≤50% without intraprocedural complications)**	133/141 (94.3%)	64/71 (90.1%)	69/70 (98.6%)	.063
**Technical success (residual stenosis ≤ 30% without intraprocedural complications)**	110/141 (78.0%)	53/71 (74.6%)	57/70 (81.4%)	.417
**In-hospital TIA**	1/141 (0.7%)	1/71 (1.4%)	0/70 (0.0%)	1.000
**In-hospital AIS**	2/140 (1.4%)	1/71 (1.4%)	1/69 (1.4%)	1.000
**In-hospital ICH**	5/141 (3.5%)	5/71 (7.0%)	0/70 (0.0%)	.058
**In-hospital death**	4/141 (2.8%)	3/71 (4.2%)	1/70 (1.4%)	.620
**In-hospital major complications (ICH, AIS or death)**	10/141 (7.1%)	9/71 (12.7%)	1/70 (1.4%)	**.017**
**Discharge mRS < 3**	112/141 (79.4%)	54/71 (76.1%)	58/70 (82.9%)	.405
**No. DWI lesions**				.256
** 0**	48/79 (60.8%)	26/38 (68.4%)	22/41 (53.7%)	
** 1-5**	19/79 (24.1%)	9/38 (23.7%)	10/41 (24.4%)	
** 6-10**	7/79 (8.9%)	1/38 (2.6%)	6/41 (14.6%)	
** >10**	5/79 (6.3%)	2/38 (5.3%)	3/41 (7.3%)	
**Last available FU**				
** Follow-up months**	9.0 [6.0–12.0] (*n* = 114)	9.0 [6.0–12.0] (*n* = 53)	11.0 [6.0–12.0] (*n* = 61)	.320
** Restenosis**	16/112 (14.3%)	4/51 (7.8%)	12/61 (19.7%)	.104
** mRS < 3**	100/118 (84.7%)	46/56 (82.1%)	54/62 (87.1%)	.609
** Δ mRS (at last FU)**				**.018** ^a^
** Improved**	49 (41.5%)	28 (50.0%)	21 (33.9%)	.093^b^
** Stable**	43 (36.4%)	13 (23.2%)	30 (48.4%)	**.007** ^b^
** Worsened**	26 (22.0%)	15 (26.8%)	11 (17.7%)	.271^b^

Percentages are column percentages. Categorical *P*-values come from Fisher’s exact test (2-sided) whenever any expected cell count < 5; otherwise from Pearson’s chi-square test. Continuous variables are reported as median [IQR]; *P*-values from Mann–Whitney *U* test. Denominators (n/N) reflect non-missing data for each subgroup and time point. Abbreviations: AIS = acute ischaemic stroke; DWI = diffusion-weighted imaging; FU = follow-up; ICH = intracranial haemorrhage; IQR = interquartile range; mRS = modified Rankin Scale; MWU = Mann–Whitney U test; N/A = not applicable; PTAS = percutaneous transluminal angioplasty and stenting; TIA = transient ischaemic attack.

^a^Pearson chi-square test (3 × 2) across Improved/Stable/Worsened vs treatment group at last FU.

^b^Per-category 2-sided Fisher’s exact test comparing Primary PTAS vs Elective at last FU (observed cases only). Bold values indicate statistically significant differences (P < .05).

Technical complications with stent deployment occurred in 2/141 patients (1.4%), both in the Primary PTAS group. Intraprocedural in-stent thrombosis was observed in 7/141 patients (5.0%), exclusively in Primary PTAS (9.9% [7/71] vs 0/70; *P* = .013). There were no intraprocedural distal emboli. One intraprocedural arterial dissection occurred in the Primary PTAS group (1/141 overall, 0.7%; 1/71 Primary PTAS, 1.4%; 0/70 Elective PTAS; *P* = 1.000). No vessel perforations were recorded. Technical success (residual stenosis ≤ 50% without intraprocedural complications) was achieved in 133/141 interventions (94.3%), with high rates in both groups (Primary 90.1% [64/71] vs Elective 98.6% [69/70]; *P* = .063; Table 3); sensitivity (residual ≤ 30%): 78.0% (74.6% [53/71] vs 81.4% [57/70]; *P* = 0.417). In-hospital TIA occurred in 1/141 patients (0.7%), AIS in 2/140 (1.4%) and ICH in 5/141 (3.5%). ICH was confined to the Primary PTAS group (7.0% [5/71] vs 0%; *P* = .058). In-hospital death occurred in 4/141 patients overall (2.8%): 3/71 patients in the Primary PTAS group (4.2%) and 1/70 patient in the Elective PTAS group (1.4%; *P* = .620). The primary safety endpoint of in-hospital major complications (ICH, AIS or death) was met in 10/141 patients (7.1%), driven by a higher rate in the Primary PTAS group (12.7% [9/71] vs 1.4% [1/70]; *P* = .017; Table 3). Regardless, at discharge, 112/141 patients (79.4%) were functionally independent, with similar proportions in Primary and Elective PTAS (76.1% vs 82.9%; *P* = .405).

Diffusion-weighted MRI within the early postprocedural period was available in 79 patients (38 Primary, 41 Elective). New ischaemic lesions were present in 31/79 (39.2%) and were predominantly fewer (≤5 lesions in 24/31, 77.4%), without significant differences in lesion burden distribution between Primary and Elective PTAS (global *P* = .256; Table 3).

### Follow-up angiographic and clinical outcomes

Serial clinical and imaging follow-up were available in most patients, with heterogeneous timing across centres; detailed distributions by predefined follow-up visits are provided in Table S4. At last available follow-up (median 9.0 months [IQR 6.0–12.0]; 9.0 [6.0–12.0] in the Primary PTAS group vs 11.0 [6.0–12.0] in the Elective PTAS group; *P* = .320), restenosis ≥50% (at any time) was documented in 16/112 patients (14.3%) overall, numerically more frequent after Elective than Primary PTAS (19.7% [12/61] vs 7.8% [4/51]; *P* = .104). Functional independence (mRS < 3) at last follow-up was achieved in 100/118 patients (84.7%), with similar proportions in the Primary and Elective PTAS groups (82.1% [46/56] vs 87.1% [54/62]; *P* = .609; Table 3).

**Figure 1:**
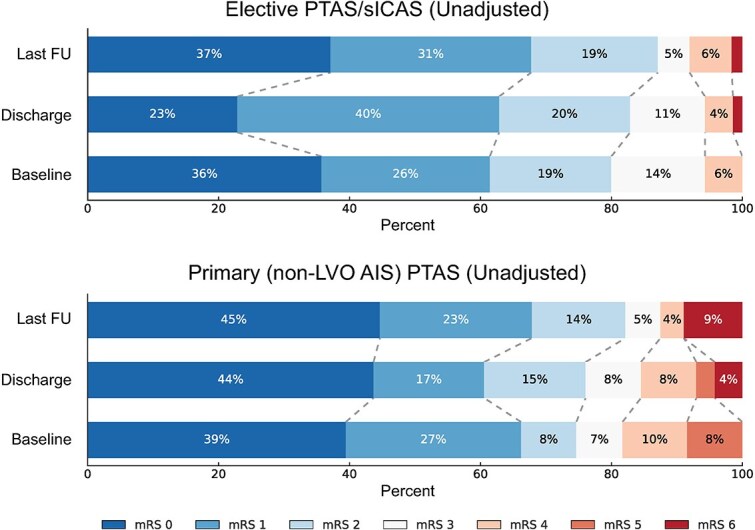
mRS evolution—Grotta bars (unadjusted). Grotta-style horizontal stacked bars show the distribution of modified Rankin scale (mRS 0–6) at 3 time points for each panel (emergent PTAS [non-LVO AIS] and elective PTAS [symptomatic]). Each bar’s n is the number of patients with an mRS recorded at that time point within the panel; segment values are within-bar percentages. “Last FU (strict)” follows FU3 → FU2 → FU1; discharge mRS is not used as last FU unless the patient died in-hospital, in which case mRS = 6 is carried forward (death treated as an absorbing state). Dashed connectors link category boundaries between adjacent time points to visualise shifts of the overall distribution; they do not represent individual patient trajectories. No imputation was performed; percentages may not sum to 100% due to rounding. Abbreviation: FU = follow-up.

Change in mRS from baseline to last follow-up differed between groups (global *P* = .018). Overall, 49/118 patients (41.5%) improved, 43/118 (36.4%) remained stable and 26/118 (22.0%) worsened. Improvement was numerically more frequent after Primary PTAS (50.0% vs 33.9%; *P* = .093), whereas stability was more common after Elective PTAS (48.4% vs 23.2%; *P* = .007), with a similar proportion of worsening (26.8% vs 17.7%; *P* = .271). The evolution of functional status in both groups is illustrated in Figure 1.

## Discussion

In this multicentre retrospective cohort, we evaluated the hydrophilic polymer–coated pEGASUS-HPC stent for sICAS used either as Primary PTAS during non-LVO AIS or as Elective PTAS for recurrent symptoms despite IMT. Overall, pEGASUS-HPC PTAS achieved substantial luminal gain, a moderate rate of restenosis, and good mid-term functional outcomes. However, periprocedural safety differed markedly by indication: major in-hospital complications occurred in 7.1% of patients overall but were almost an order of magnitude more frequent after Primary than Elective PTAS, whereas functional independence and restenosis at last follow-up were similar between groups. This comparison should therefore be interpreted as an indication-based descriptive contrast rather than as a causal comparison of interchangeable treatment strategies. These findings suggest that pEGASUS-HPC PTAS can be performed with acceptable safety in carefully selected sICAS patients, particularly in the elective setting, whereas the hyperacute AIS environment remains high-risk even with coated technology.

### Relation to RCTs and guideline recommendations

Randomised trials have defined both the natural history of sICAS and the hazards of first-generation intracranial stenting. In WASID, patients with 70%–99% intracranial stenosis had a one-year ipsilateral stroke risk approaching 20% despite medical therapy.^1^ SAMMPRIS and VISSIT subsequently showed that PTAS with the Wingspan self-expanding stent or a balloon-expandable stent was inferior to IMT alone, with 30-day stroke or death rates of 14.7% and 24.1%, respectively, in the stenting arms.^2^^,^^15^ More recently, CASSISS demonstrated that with refined selection criteria, delayed intervention and experienced operators, Wingspan PTAS could be performed with a lower early event rate (5.1% 30-day stroke or death), but again without benefit over IMT alone.^3^ These trials underpin current recommendations that IMT should be first-line treatment and that intracranial stenting, if considered, should be reserved for highly selected, medically refractory patients.^4^

Accordingly, the overall 7.1% major complication rate in our cohort compares favourably with SAMMPRIS and VISSIT and is broadly consistent with contemporary series and meta-analyses of self-expanding stents, where periprocedural stroke or death rates around 6% have been reported.^8^^,^^16^ However, we found distinct risk profiles: the 12.7% rate of major complications after Primary PTAS approaches the early hazard of the RCTs,^2^^,^^15^ whereas the 1.4% rate after Elective PTAS is close to the on-label Wingspan experience in WEAVE/WOVEN (2.6% periprocedural events).^17^ These gradients reinforce that timing, clinical context and selection remain key determinants of intracranial stenting risk, even with newer coated devices.

### Primary versus elective pEGASUS-HPC PTAS

The contrast between Primary and Elective PTAS likely reflects differences in both patient biology and procedural conditions rather than an intrinsic device effect. Primary PTAS was performed during or shortly after AIS, often in the setting of unstable haemodynamics, fresh thrombus and incomplete antiplatelet preloading. Balloon angioplasty under these circumstances may destabilise plaque and expose thrombogenic surfaces, and acute stent deployment in a prothrombotic milieu favours in-stent thrombosis despite the antithrombogenic coating.^18^^,^^19^ In our series, all intraprocedural in-stent thromboses occurred in the Primary PTAS group and were accompanied by higher rates of ICH and early major complications. This may be partly explained by the AIS setting: rapid restoration of flow into previously ischaemic, blood–brain–barrier–disrupted tissue increases the risk of haemorrhagic transformation and reperfusion injury, particularly in the context of intensive antithrombotic therapy.^20^ By contrast, Elective PTAS was undertaken after optimisation of IMT, with more stable symptoms, standardised dual antiplatelet regimens, and greater opportunity for procedural planning; in this setting, periprocedural risk was lower, technical success was numerically higher, and mid-term functional outcomes were comparable to those achieved with Primary PTAS. Technical success (residual stenosis ≤ 50% without intraprocedural complications) was high overall, although numerically lower in Primary than Elective PTAS (90.1% vs 98.6%; *P* = .063), and between-group differences were driven mainly by intraprocedural events rather than inadequate luminal gain. These observations are consistent with prior work showing that carefully selected, delayed stenting in experienced centres can be performed at lower risk than early intervention.^3^^,^^4^^,^^8^^,^^16^ Our findings should not be interpreted as supporting routine Primary PTAS in the acute setting. Rather, they add real-world safety information for selected acute non-LVO AIS presentations and support restricting acute use to rescue scenarios with a compelling haemodynamic or recurrent-symptom rationale despite optimised acute medical management.

### Coated stents, restenosis and comparison with other technologies

The pEGASUS-HPC stent is part of a broader class of surface-modified neurovascular implants designed to reduce thrombogenicity, building on in vitro and aneurysm data showing reduced thrombin generation, platelet activation and thromboembolic events with HPC devices.^9–12^ In a single-centre pEGASUS-HPC ICAS series (*n* = 42), stenosis reduction was comparable to ours but overall complications were higher (16.3%), largely reflecting the predominance of emergency AIS procedures.^21^ A multicentre comparison of pEGASUS-HPC with the fibrin–heparin–coated CREDO heal stent in acute ICAS, together with a multicentre CREDO heal rescue series, also found high technical success and acceptable early safety for both devices.^13^^,^^22^ Taken with our data, these studies support coated intracranial stents as a useful option for acute and elective sICAS, while emphasising the need for careful selection and optimised antithrombotic management.

In-stent restenosis is another major concern. Meta-analyses of self-expanding intracranial stents report restenosis rates around 13%–20%, with higher rates for Wingspan than for off-label low-profile stents.^8^^,^^16^ Drug-eluting intracranial stents further reduce ISR: in the NOVA RCT, drug-eluting stents achieved a one-year ISR rate of about 9.5% versus 30.2% with bare-metal stents, with parallel reductions in recurrent ischaemia,^23^ while a subsequent meta-analysis confirmed the restenosis advantage of drug-eluting over bare-metal stents.^7^ In our cohort, restenosis occurred in 14.3% of patients with angiographic follow-up at a median of 9 months, within the lower range reported for self-expanding stents,^8^^,^^16^ though interpretation is limited by heterogeneous, non–core lab follow-up. Our data do not demonstrate a clear restenosis advantage of pEGASUS-HPC over other self-expanding stents, but suggest that its durability is at least comparable to contemporary devices.

### Balloon angioplasty alone as an alternative

Balloon angioplasty alone, often performed in a submaximal fashion, has re-emerged as an alternative to PTAS, aiming to reduce device-related complications at the expense of higher residual stenosis. A meta-analysis of submaximal angioplasty reported a 30-day stroke or death rate of about 5%, low long-term stroke rates, and an 18.4% restenosis rate, suggesting a favourable safety profile compared with early stenting experience.^24^ The recently published BASIS RCT showed that balloon angioplasty plus IMT reduced 1-year recurrent stroke or TIA compared with IMT alone in selected patients with sICAS, supporting the concept that carefully performed angioplasty can improve outcomes over medical therapy alone in appropriately chosen cases.^6^ In our Elective cohort, pEGASUS-HPC PTAS achieved low major complication rates and substantially lower residual stenosis than typically reported after balloon angioplasty alone, which may be relevant for flow-limiting lesions. However, we did not include a balloon-only arm, and our data cannot determine whether the additional luminal gain from stenting translates into superior long-term stroke prevention. Because pre-dilation was performed in nearly all cases, the observed angiographic and clinical outcomes should be interpreted as reflecting an overall PTAS strategy with balloon lesion preparation followed by pEGASUS-HPC implantation, rather than the isolated effect of the stent alone. Future comparative studies, ideally randomised or at least carefully matched registries, are needed to clarify the relative roles of coated stents, drug-eluting stents and angioplasty-only strategies.

### Diffusion-weighted lesions and “silent” ischaemia

New DWI lesions were detected in almost 40% of patients with early postprocedural MRI, typically as a small number of punctate infarcts and usually without clear clinical correlates.^25^ This frequency is somewhat lower but conceptually similar to reports of clinically silent microinfarcts after treatment of unruptured intracranial aneurysms with HPC flow diverters, where DWI lesions were observed in more than half of patients despite good clinical outcomes.^26^ These data suggest that, even with surface-modified devices, microembolic phenomena remain common after intracranial endovascular procedures, and the burden of “silent” ischaemia should be considered when comparing different strategies for sICAS.^27^

### Limitations

This retrospective, observational study relied on treatment decisions (use and timing of Primary vs Elective PTAS, antithrombotic regimens) made at the discretion of local teams across 18 centres, which introduces selection bias, confounding and procedural heterogeneity. Periprocedural management was not standardised, including balloon strategy, post-dilation and adjunctive medications, so some thrombotic or haemorrhagic events may reflect the overall PTAS strategy or operator-specific practice rather than the stent itself. Because most patients underwent pre-dilation, complications could not always be definitively attributed to balloon angioplasty versus stent deployment. Clinical and imaging follow-up were incomplete, varied in timing and modality, and were not core-lab adjudicated, limiting the precision of our estimates of in-stent restenosis, late events and new DWI lesions, which were only systematically assessed in a subset of patients. The ΔmRS analysis is additionally limited by differences in baseline timing: in Primary PTAS, baseline mRS generally reflected the acute stroke presentation, whereas in Elective PTAS it reflected preprocedural status after the qualifying event and possible interval recovery. Although this is, to our knowledge, the largest reported pEGASUS-HPC series in sICAS, the sample size remains modest for subgroup analyses and infrequent outcomes, and the absence of a medical-therapy-only or balloon-only comparator arm precludes any conclusions about the superiority of pEGASUS-HPC PTAS over non-stent strategies.

## Conclusion

In this 18-centre retrospective cohort, pEGASUS-HPC PTAS for sICAS achieved substantial luminal gain, moderate restenosis rates, and generally good mid-term functional outcomes. Periprocedural safety, however, was strongly context-dependent: Elective PTAS in medically refractory, non-acute patients was associated with very low rates of major complications, whereas Primary PTAS during non-LVO AIS carried a significantly higher risk of intraprocedural thrombosis, haemorrhage and early serious events. These findings support using pEGASUS-HPC mainly as an elective option in carefully selected sICAS patients treated in experienced centres, while restricting primary use in the hyperacute setting to rescue scenarios with a clear haemodynamic rationale and robust antithrombotic protocols.

## Supplementary Material

Supplementary_File_aakag091

## Data Availability

The datasets generated and analysed during the current study are available from the corresponding author on reasonable request in anonymised form.
