# Supplementary material for: Primary versus elective percutaneous transluminal angioplasty with stenting for symptomatic intracranial atherosclerotic stenosis: a 18-centre retrospective cohort study using the pEGASUS-HPC stent
Source: Eur Stroke J. 2026 Jul 31;11(7):aakag091. doi: 10.1093/esj/aakag091 (PMC13425413; doi:10.1093/esj/aakag091)
Supplement: Supplementary_File_aakag091 [file supplementary_file_aakag091.docx]

**SUPPLEMENTARY MATERIAL**

**Table S1.** Balloon-related procedural details by treatment group

| **Variable** | **Overall** | **Primary PTAS (AIS), n=71** | **Elective PTAS (symptomatic), n=70** | **P-value** |
| --- | --- | --- | --- | --- |
| Balloon pre-dilation | 137 (97.2) | 70 (98.6) | 67 (95.7) | 0.366 |
| (Pre-dilation) Balloon 1 type | 137 | 70 | 67 | **<0.001** |
| NeuroSpeed | 39 (28.5) | 30 (42.9) | 9 (13.4) |  |
| pITA | 57 (41.6) | 22 (31.4) | 35 (52.2) |  |
| Maverick | 2 (1.5) | 0 (0.0) | 2 (3.0) |  |
| NC Emerge | 1 (0.7) | 0 (0.0) | 1 (1.5) |  |
| SeQuent | 11 (8.0) | 3 (4.3) | 8 (11.9) |  |
| Elutax | 2 (1.5) | 1 (1.4) | 1 (1.5) |  |
| Ryujin | 2 (1.5) | 1 (1.4) | 1 (1.5) |  |
| Aviator | 1 (0.7) | 1 (1.4) | 0 (0.0) |  |
| Gateway | 1 (0.7) | 1 (1.4) | 0 (0.0) |  |
| NC trek | 2 (1.5) | 0 (0.0) | 2 (3.0) |  |
| Prevail | 2 (1.5) | 0 (0.0) | 2 (3.0) |  |
| Ryurei | 17 (12.4) | 11 (15.7) | 6 (9.0) |  |
| Second balloon for pre-dilation | 50 (35.5) | 14 (19.7) | 36 (51.4) | **<0.001** |
| (Pre-dilation) Balloon 2 type | 50 | 14 | 36 | **0.001** |
| NeuroSpeed | 3 (6.0) | 3 (21.4) | 0 (0.0) |  |
| pITA | 13 (26.0) | 4 (28.6) | 9 (25.0) |  |
| Maverick | 1 (2.0) | 1 (7.1) | 0 (0.0) |  |
| SeQuent | 16 (32.0) | 1 (7.1) | 15 (41.7) |  |
| Elutax | 6 (12.0) | 0 (0.0) | 6 (16.7) |  |
| NC trek | 2 (4.0) | 0 (0.0) | 2 (5.6) |  |
| Ryurei | 9 (18.0) | 5 (35.7) | 4 (11.1) |  |
| Post-dilation | 10 (7.1) | 7 (9.9) | 3 (4.3) | 0.326 |
| (Post-dilation) Balloon 1 type |  |  |  | 0.450 |
| NeuroSpeed | 3 (30.0) | 3 (42.9) | 0 (0.0) |  |
| pITA | 4 (40.0) | 2 (28.6) | 2 (66.7) |  |
| Maverick | 1 (10.0) | 1 (14.3) | 0 (0.0) |  |
| Elutax | 1 (10.0) | 0 (0.0) | 1 (33.3) |  |
| Ryurei | 1 (10.0) | 1 (14.3) | 0 (0.0) |  |
| DEB for pre-dilation | 29 (20.6) | 4 (5.6) | 25 (35.7) | **<0.001** |
| DEB for post-dilation | 1 (10.0) | 0 (0.0) | 1 (33.3) | 0.300 |

Values are median [IQR] or n (column %). Primary PTAS = procedures performed during/after AIS (rescue); Elective PTAS = symptomatic elective procedures. P-values: Mann–Whitney U for continuous; Pearson χ² or Fisher’s exact as appropriate for categorical (small expected counts). Percentages use each column’s non-missing denominator; “Overall” uses the variable’s non-missing denominator. Overall percentages are calculated using the denominator shown in the “Overall” column. For balloon-related variables, denominators are restricted to those undergoing the corresponding step: Balloon 1 N=137 (Primary PTAS 70; Elective 67), Balloon 2 N=50 (14; 36), and post-dilation type/DEB post-dilation N=10 (7; 3). Abbreviations: AIS, acute ischemic stroke; DEB, drug-eluting balloon; PTAS, percutaneous transluminal angioplasty and stenting.

**Table S2.** Antiplatelet treatment details

| **Variable / Category** | **Overall (N=141)** | **Primary PTAS (n=71)** | **Elective PTAS (n=70)** | **P-value** |
| --- | --- | --- | --- | --- |
| Antiplatelet (prior treatment) |  |  |  | **<0.001** |
| • SAPT | 34 (30.4) | 23 (54.8) | 11 (15.7) |  |
| • DAPT | 78 (69.6) | 19 (45.2) | 59 (84.3) |  |
| Antiplatelet loading (yes/) | 82 (58.2) | 41 (57.7) | 41 (58.6) | 0.921 |
| Antiplatelet loading (drugs) |  |  |  | **<0.001** |
| • AAS 100 mg + ticagrelor 180 mg | 23 (28.0) | 0 (0.0) | 23 (56.1) |  |
| • AAS 500 mg + ticagrelor 180 mg | 9 (11.0) | 8 (19.5) | 1 (2.4) |  |
| • AAS 300 mg + ticagrelor 270 mg | 2 (2.4) | 2 (4.9) | 0 (0.0) |  |
| • AAS 500 mg + ticagrelor 180 mg + eptifibatide | 10 (12.2) | 10 (24.4) | 0 (0.0) |  |
| • AAS 100 mg + prasugrel 10 mg | 5 (6.1) | 0 (0.0) | 5 (12.2) |  |
| • Ticagrelor 180 mg | 11 (13.4) | 9 (22.0) | 2 (4.9) |  |
| • Clopidogrel 300 mg | 1 (1.2) | 1 (2.4) | 0 (0.0) |  |
| • Tirofiban 50 mcg | 1 (1.2) | 1 (2.4) | 0 (0.0) |  |
| • AAS 500 mg | 8 (9.8) | 1 (2.4) | 7 (17.1) |  |
| • AAS 500 mg + tirofiban | 7 (8.5) | 6 (14.6) | 1 (2.4) |  |
| • Cangrelor (bolus/weight) | 1 (1.2) | 1 (2.4) | 0 (0.0) |  |
| • AAS 325 mg + prasugrel 40 mg | 1 (1.2) | 0 (0.0) | 1 (2.4) |  |
| • Prasugrel 60 mg | 2 (2.4) | 1 (2.4) | 1 (2.4) |  |
| • AAS 500 mg + clopidogrel 600 mg | 1 (1.2) | 1 (2.4) | 0 (0.0) |  |
| Postprocedural antiplatelet scheme |  |  |  | **0.001** |
| • SAPT | 14 (9.9) | 13 (18.3) | 1 (1.4) |  |
| • DAPT | 127 (90.1) | 58 (81.7) | 69 (98.6) |  |
| Postprocedural antiplatelet (drugs) |  |  |  | **<0.001** |
| • Ticagrelor | 11 (7.8) | 10 (14.1) | 1 (1.4) |  |
| • Prasugrel | 3 (2.1) | 3 (4.2) | 0 (0.0) |  |
| • AAS + clopidogrel | 25 (17.7) | 18 (25.4) | 7 (10.0) |  |
| • AAS + ticagrelor | 82 (58.2) | 33 (46.5) | 49 (70.0) |  |
| • AAS + prasugrel | 20 (14.2) | 7 (9.9) | 13 (18.6) |  |

Values are counts and percentages. Primary PTAS = procedures performed during/after AIS (rescue); Elective PTAS = symptomatic elective procedures. P-values: Pearson χ² or Fisher’s exact as appropriate for categorical (small expected counts). Percentages use each column’s non-missing denominator; “Overall” uses the variable’s non-missing denominator. Missing values: antiplatelet premedication scheme (n=29), antiplatelet loading drugs (n=59); all other variables 0.

**Table S3.** Exploratory outcomes in Primary PTAS patients according to postprocedural antiplatelet regimen

| **Outcome** | **Postprocedural SAPT, n=13** | **Postprocedural DAPT, n=58** | **p-value** |
| --- | --- | --- | --- |
| Post-treatment residual stenosis, median [IQR] | 25.6 [18.5–30.0] (n=13) | 25.0 [15.8–30.0] (n=58) | 0.840 |
| Technical success (residual ≤50% + no intraprocedural complications) | 11/13 (84.6%) | 53/58 (91.4%) | 0.604 |
| Intraprocedural in-stent thrombosis | 2/13 (15.4%) | 5/58 (8.6%) | 0.604 |
| Intraprocedural distal embolism | 0/13 (0.0%) | 0/58 (0.0%) | — |
| Postprocedural in-hospital TIA | 1/13 (7.7%) | 0/58 (0.0%) | 0.183 |
| Postprocedural in-hospital AIS | 0/13 (0.0%) | 1/58 (1.7%) | 1.000 |
| Any new DWI lesion | 4/8 (50.0%)† | 8/30 (26.7%)† | 0.232 |
| No. DWI lesions (0 / 1–5 / 6–10 / ≥10) | 4/8 / 3/8 / 0/8 / 1/8† | 22/30 / 6/30 / 1/30 / 1/30† | 0.272‡ |
| Restenosis ≥50% at any follow-up | 1/9 (11.1%)§ | 3/42 (7.1%)§ | 0.552 |
| In-hospital major complications (ICH, AIS, or death) | 1/13 (7.7%) | 8/58 (13.8%) | 1.000 |

† DWI denominators reflect available DWI data only (SAPT n=8; DAPT n=30). ‡ Mann–Whitney (ordinal) on the 4-level category variable (exact p). § Restenosis denominators reflect any FU restenosis data available (SAPT n=9; DAPT n=42).

**Table S4.** Follow-up outcomes by timepoint

| **Outcome** | **Overall** | **Primary PTAS (AIS), n=71** | **Elective PTAS (symptomatic), n=70** | **P-value** |
| --- | --- | --- | --- | --- |
| **FU1** |  |  |  |  |
| • Follow-up months (FU1) (median [IQR]) | 5.0 [3.0–6.0] (N=103) | 6.0 [3.0–6.0] (N=49) | 4.0 [3.0–6.0] (N=54) | 0.132 |
| • FU1 mRS <3 | 92/110 (83.6%) | 44/53 (83.0%) | 48/57 (84.2%) | 1.000 |
| • Restenosis at FU1 | 13/102 (12.7%) | 4/48 (8.3%) | 9/54 (16.7%) | 0.246 |
| **FU2** |  |  |  |  |
| • Follow-up months (FU2) (median [IQR]) | 12.0 [10.0–12.0] (N=65) | 12.0 [10.0–12.0] (N=28) | 12.0 [10.0–12.0] (N=37) | 0.591 |
| • FU2 mRS <3 | 56/69 (81.2%) | 25/33 (75.8%) | 31/36 (86.1%) | 0.360 |
| • Restenosis at FU2 | 7/65 (10.8%) | 1/28 (3.6%) | 6/37 (16.2%) | 0.130 |
| **FU3** |  |  |  |  |
| • Follow-up months (FU3) (median [IQR]) | 20.0 [20.0–21.0] (N=13) | 19.5 [19.0–22.0] (N=4) | 21.0 [20.0–21.0] (N=9) | 0.336 |
| • FU3 mRS <3 | 13/19 (68.4%) | 4/9 (44.4%) | 9/10 (90.0%) | 0.057 |
| • Restenosis at FU3 | 1/13 (7.7%) | 0/4 (0.0%) | 1/9 (11.1%) | 1.000 |
| **Last FU** |  |  |  |  |
| • Follow-up months (last FU) (median [IQR]) | 9.0 [6.0–12.0] (N=114) | 9.0 [6.0–12.0] (N=53) | 11.0 [6.0–12.0] (N=61) | 0.320 |
| • Last FU (strict) mRS <3 | 100/118 (84.7%) | 46/56 (82.1%) | 54/62 (87.1%) | 0.609 |
| • Restenosis (ever up to last FU) | 16/112 (14.3%) | 4/51 (7.8%) | 12/61 (19.7%) | 0.104 |

Values are median [IQR] or n (column %). Categorical p-values use two-sided Fisher’s exact test when any expected cell count is <5; otherwise Pearson’s χ². Continuous variables are summarized as median [IQR]; p-values from Mann–Whitney U. Denominators (n/N) reflect non-missing data for each subgroup and timepoint (observed cases).
